# Neuroprotective Effects of Adenosine A1 Receptor Signaling on Cognitive Impairment Induced by Chronic Intermittent Hypoxia in Mice

**DOI:** 10.3389/fncel.2020.00202

**Published:** 2020-07-09

**Authors:** Yichun Zhang, Hongchao Cao, Xuehao Qiu, Danfen Xu, Yifeng Chen, Gregory N. Barnes, Yunjia Tu, Adwoa Takyiwaa Gyabaah, Abdulla Husain Abdulla Ahmed Gharbal, Chenlei Peng, Jun Cai, Xiaohong Cai

**Affiliations:** ^1^Department of Pediatrics, The Second Affiliated Hospital and Yuying Children’s Hospital, Wenzhou Medical University, Wenzhou, China; ^2^Department of Internal Medicine, Hwa Mei Hospital, University of Chinese Academy of Sciences (Ningbo No. 2 Hospital), Ningbo, China; ^3^Department of Neurology, University of Louisville School of Medicine, Louisville, KY, United States; ^4^Department of Pediatrics, Pediatric Research Institute, University of Louisville School of Medicine, Louisville, KY, United States

**Keywords:** chronic intermittent hypoxia, cognitive dysfunction, adenosine A1 receptor, synaptic plasticity, PKC, neuroprotection

## Abstract

Obstructive sleep apnea-hypopnea syndrome (OSAHS) is a breathing disorder associated with cognitive impairment. However, the mechanisms leading to cognitive deficits in OSAHS remain uncertain. In this study, a mouse model of chronic intermittent hypoxia (CIH) exposures were applied for simulating the deoxygenation-reoxygenation events occurring in OSAHS. The conventional adenosine A1 receptor gene (*A1R*) knockout mice and the A1R agonist CCPA- or antagonist DPCPX-administrated mice were utilized to determine the precise function of A1R signaling in the process of OSAHS-relevant cognitive impairment. We demonstrated that CIH induced morphological changes and apoptosis in hippocampal neurons. Further, CIH blunted hippocampal long-term potentiation (LTP) and resulted in learning/memory impairment. Disruption of adenosine A1R exacerbated morphological, cellular, and functional damage induced by CIH. In contrast, activation of adenosine A1R signaling reduced morphological changes and apoptosis of hippocampal neurons, promoted LTP, and enhanced learning and memory. A1Rs may up-regulate protein kinase C (PKC) and its subtype PKC-ζ through the activation of Gα(i) improve spatial learning and memory disorder induced by CIH in mice. Taken together, A1R signaling plays a neuroprotective role in CIH-induced cognitive dysfunction and pathological changes in the hippocampus.

## Introduction

Childhood obstructive sleep apnea-hypopnea syndrome (OSAHS) is a disease characterized by repeated episodes of upper airway collapse during sleep which causes hypopneas/apneas that affect children’s health and growth. It can occur at any age group, especially in pre-school children, having a prevalence of between 1.2% and 5.7% of the general population (Marcus et al., 2012). OSAHS has been implicated in respiratory, cardiovascular, endocrine, digestive, genitourinary, muscular, and nervous system damage in adults. In children, it more commonly results in learning and memory deficits (Xie and Yung, 2012; Yang et al., 2013). Chronic intermittent hypoxia (CIH) is a featured characteristic of OSAHS, which plays a cardinal role in impairments in synaptic plasticity and neurocognition. Recently, we and others reported that cognitive deficits and apoptosis of hippocampal CA1 neurons occur in rodents following CIH exposure (Cai et al., 2014; Wang et al., 2015; Yuan et al., 2015). Accumulating evidence also shows that CIH exposures suppress long-term potentiation (LTP) in hippocampal neurons (Payne et al., 2004; Xie et al., 2010; Wall et al., 2014; Khuu et al., 2019). Taken together, these findings suggest that CIH is involved in the cognitive impairment of OSAHS. However, the precise mechanism (s) underlying the OSAHS-relevant cognitive dysfunction remains unclear.

Adenosine is an inhibitory neuromodulator in the central nervous system (CNS; Fredholm et al., 2005). Adenosine receptors are members of the G-protein coupled receptor family and have been classified as A1, A2A, A2B, and A3. Hypoxia-induced accumulation of extracellular adenosine causes an activity-dependent down-regulation of adenosine A1 receptors (A1Rs; Coelho et al., 2006; Chen Z. et al., 2014). Activation of A1RS plays an important role in neuroprotection predominantly by inhibiting synaptic transmission (Cunha, 2001). A1R activation reduces neuronal excitability and energy consumption, thereby improving neuronal tolerance to hypoxia (Giust et al., 2014; Duarte et al., 2016). A1Rs are expressed throughout the body and the CNS and are found in the cerebral cortex, hippocampus, cerebellum, thalamus, brainstem, and spinal cord (Giménez-Llort et al., 2005). These receptors have the highest affinity for adenosine, link with Gα(i) protein (Baker and Hill, 2007; Crawford et al., 2011; Draper-Joyce et al., 2018), and signal *via* adenylate cyclase (AC)—cyclic adenosine monophosphate (cAMP)—protein kinase C (PKC) pathways (Okada et al., 2001; Di-Capua et al., 2003; Rombo et al., 2016). Accumulating evidence indicates that A1Rs are tightly associated with cognitive impairments and neural plasticity (Chen J.-F. et al., 2014; Chen, 2014). Several studies have also shown that activation of A1Rs has neuroprotective effects in a mouse hypoxic-ischemic brain injury model (Zamani et al., 2013; Tregub et al., 2014). However, detrimental effects were reported in other animal studies (Mioranzza et al., 2011; Chiu et al., 2012; Düster et al., 2014). Olsson et al. (2004) found that genetic deletion of A1R in mice does not alter the extent of the brain or neuronal ischemic lesion. The reason for this can only be speculated upon, the deletion may have induced processes in the brain preventing the detrimental consequences of A1R loss (Olsson et al., 2004). These discrepant findings suggest a complex role of A1Rs in the CNS, which may be involved in different signaling pathways underlying the pathogenesis or progression of the disease. A1Rs have been identified as a key signaling pathway that mediates brain preconditioning (Heurteaux et al., 1995; Plamondon et al., 1999; Reshef et al., 2000; Hiraide et al., 2001), however their prolonged activation desensitizes adenosine responses (Coelho et al., 2006; Chen Z. et al., 2014). Francisco Cayabyab’s lab showed that prolonged A1R activation during hypoxia or ischemia could contribute significantly to increased neuronal death by cause clathrin-mediated GluA2 and GluA1 AMPAR endocytosis and persistent synaptic depression (Chen Z. et al., 2014). Timing is essential to determine when A1R activation plays a neuroprotective role. It has been reported that A1R activation attenuates brain damage when occurring shortly before or simultaneously during brain insult, but A1Rs may lose their neuroprotective effects when activated after brain insult (Lubitz, 1999; Mendonça et al., 2000; Lubitz et al., 2001).

Adenosine is one of many metabolites whose production is increased under hypoxia. Our previous study has shown that, in children with moderate-to-severe OSAHS and cognitive dysfunction, morning plasma adenosine levels were markedly high (Yan et al., 2013). Given the important role of A1Rs in CNS diseases, we hypothesize that A1R signaling plays an important role in the process of OSAHS-relevant cognitive impairment. Thus, a mouse model of CIH exposures was applied for simulating the deoxygenation-reoxygenation events occurring in OSAHS. The precise function of A1R signaling was investigated *via* the administration of A1R agonists and antagonists to wild-type mice and using the conventional *A1R* gene knockout mouse.

## Materials and Methods

### Reagents

Dimethyl sulfoxide (DMSO) was obtained from Amresco Incorporation (USA); 2-chloro-N (6)-cyclopentyl-adenosine (CCPA), 8-cyclopentyl-1,3-dipropylxanthine (DPCPX), quick-hardening mounting medium, and mouse anti-β-tubulin antibody (Cat. T8328) were purchased from Sigma Corporation (USA); Hematoxylin and Eosin (HE) staining kit was obtained from Beyotime Institute of Biotechnology (China); 3,3-diaminobenzidine (DAB) was purchased from ZSGB-BIO Corporation (China); Bicinchoninic acid (BCA) protein assay Kit and ECL western blotting detection reagent were purchased from Pierce Corporation (USA); TUNEL assay kit was from Roche Limited (USA); PCR primer was obtained from Invitrogen Company (USA); Rabbit anti-caspase-3 antibody (Cat. 9662), HRP-labeled goat anti-rabbit secondary antibody (Cat. 7074), and HRP-labeled horse anti-mouse secondary antibody (Cat. 7076) were purchased from Cell Signal Technology (USA); Rabbit anti-Syntaxin (Cat. ab188583), rabbit anti-PKC (Cat. ab19031), rabbit anti-PKC-ζ (Cat. ab59364), and rabbit anti-Gα (i; Cat. ab58916) antibodies were obtained from Abcam Company (USA). Mouse anti-β-tubulin antibody was purchased from Beyotime Biotechnology (China).

### Animals

All animal use procedures were as per the National Institute of Health Guide for the Care and Use of Laboratory Animals and approved by the Ethics Committee of Wenzhou Medical University. Four-week-old C57BL/6NJ male mice, weighing 12–20 g, were purchased from Shanghai Laboratory Animal Center (China). *A1R* conventional knockout mice (*A1R*−/−; Johansson et al., 2001) were obtained from the Jackson Laboratory (USA) and bred on site. The genotype of *A1R*−/− mice were confirmed by standard PCR with the following primers: Forward 5′-TACTTCAACTTCTTCGTCTGGGT-3′ and Reverse 5′-CTTGTGGATTCGGAAGGCATAGA-3′ for wild-type band (339 bp); Forward 5′- GAATTCTTGAAGACGAAAGG-3′ and Reverse 5′-AAGGCTGAGGAGGAACAGTG-3′ for mutant band (200 bp).

### Experimental Groups

A total of 36 C57BL/6NJ wild-type male mice were randomly divided into six groups: room air control group (A), intermittent air control group (AC), CIH group (IH), CIH treated with A1R agonist CCPA group (CCPA), CIH treated with A1Rs antagonist DPCPX group (DPCPX), and CIH treated with DMSO solvent control group (DMSO). Six *A1R−/−* male mice randomly selected from 10 *A1R* knockout mice (4 weeks old) were assigned for CIH exposures (KO). Chronic treatment with the selective AR1 agonist CCPA enhances spatial learning and memory in C57BL/6 mice (Von Lubitz et al., 1993), and A1R KO mice show normal acquisition and retention of spatial reference memory as well as spatial working memory (Giménez-Llort et al., 2002, 2005; Lang et al., 2003). Thus we did not recruit A and AC groups with CCPA, DPCPX, or *A1R* deletion (KO) as additional controls in this study.

### Administration of A1R Agonist and Antagonist

The mice in the CCPA, DPCPX, and DMSO groups were injected intraperitoneally with either CCPA (0.002 mg in 100 μl PBS containing 5% DMSO, 5 μl/g body weight), DPCPX (0.01 mg in 100 μl PBS containing 5% DMSO, 5 μl/g body weight), or DMSO (PBS containing 5% DMSO, 5 μl/g bodyweight) just before daily CIH exposures.

### Chronic Intermittent Hypoxia Exposures

The mouse model of CIH was developed and reported previously (Wang et al., 2011) with modifications. Mice were placed in the intermittent hypoxia chamber, a computer-controlled nitrogen/oxygen gas delivery system (Scientific Research Center of Wenzhou Medical College, Zhejiang, China), to produce hypoxia-reoxygenation episodes following the protocol previously reported (Cai et al., 2014). Briefly, O_2_ concentration in the intermittent hypoxia chamber fluctuated from 21.0 ± 0.5% to a nadir of 8.0% ± 1.5% (30 s for 8.0% O_2_ and 18 s for 21.0% O_2_) in 90 s per cycle, 40 cycles per hour, 7 h a day during the light cycle, for a total 4 weeks. Ambient temperature was kept at 22 ± 2°C. The AC group had the same treatment conditions as CIH groups except that they were exposed to the compressed air, and the A group were exposed to room air.

### 8-arm (4-arm Baited) Radial Maze Test

Spatial memory was assessed using the eight-arm radial maze with four baited arms as described in our previous report (Cai et al., 2014). The maze was located in a testing room maintained at 22 ± 2°C, the humidity of 50–70%, and 12 h light/dark cycle. During the entire experiment, six male mice in each experimental group were fed a quantitative diet to maintain their body weight at 80–85% of their free-feeding weight, but water was offered *ad libitum*. This test was accomplished without harm or pain to the mice except for the injections of the drug at the same frequency. We recorded the body weight of mice and observed the spirit, diet, and activity of mice in the experiment process. The experiment was conducted between 17:00 and 20:30 daily. Throughout the entire training and testing sessions, food pellets were provided in arms 2, 4, 6, and 8 only. Each mouse was placed at the center of the maze with all arm entries closed. Fifteen seconds later, the entries of all arms were opened and the mouse was allowed to explore freely. The training was ended after all the pellets were consumed, or after 5 min, depending on which one occurred first. A correct choice occurred if the mouse chose an arm with food and consumed it. Other choices were considered as wrong and noted as the total errors (TE). When a mouse revisited the arms in which the bait had been obtained before, this was scored as working memory errors (WME). Entry into the arm without the bait was scored as reference memory errors (RME). The time for the mouse to complete the training was recorded as the total time (TT). Mice were subjected to 28 days of H/R and the behavioral training started on day 21 to day 27 after daily CIH exposures. All mice were trained for 7 days. The 8-arm radial maze test was performed immediately after the last hypoxia-reoxygenation cycle and initiation on day 28 The test of radial maze performance. and WME, RME, TE, and TT were recorded.

### Brain Tissue Collection

Following the entire CIH procedure or 8-arm radial maze test, mice were anesthetized with 2% sodium pentobarbital (45 mg/kg body weight) by intraperitoneal injection. Then brains were dissected into an ice-cold cutting solution (sucrose 225 mM, KCl 2.5 mM, NaH_2_PO_4_ 1.25 mM, NaHCO_3_ 28 mM, glucose 7 mM, CaCl_2_•2H_2_O 0.5 mM, MgCl_2_•6H_2_O 7 mM) at 4°C in 95% O_2_, 5% CO_2_ gas mixture. The right and left brain hemispheres were separated along the midline. The right hemispheres from six mice each group were used for electrophysiology to detect LTP. The left hemispheres were either fixed in 4% paraformaldehyde for HE staining and TUNEL assay or dissected to collect flash-frozen hippocampi for Western blots.

### Hippocampal Slice Preparation and LTP Measurement

The right hemisphere was quickly removed, rinsed in chilled artificial cerebrospinal fluid (aCSF: NaCl 125 mM, KCl 2.5 mM, NaH_2_PO_4_ 1.25mM, NaHCO_3_ 25 mM, glucose 25 mM, CaCl_2_•2H_2_O 2 mM, MgCl_2_•6H_2_O 1 mM, pH: 7.35–7.45), and mounted for vibratome sectioning. The coronal cortico-hippocampal slices were prepared at 300 μm thickness and allowed to recover for at least 1 h in a chamber perfused continuously with warm (33°C), oxygenated (95% O_2_/5% CO_2_), aCSF at 1 ml/min. Then, slices were transferred to the recording chamber, which was superfused with aCSF aerated with carbogen (95% O_2_/5% CO_2_). The aCSF-filled recording electrode (2–3 MΩ) was placed in the CA1 pyramidal cell body layer, and the stimulating electrode was placed onto the Schaffer collateral pathway. The extracellular recordings of the field excitatory postsynaptic potential (fEPSP) in the pyramidal cell body layer were obtained from the recording electrode. The stimulus to elicit 40–50% of the maximum fEPSP was used for the baseline recordings. The pre-LTP baseline fEPSP response to a test pulse was measured and recorded every minute for 30 min after the baseline had stabilized. LTP was then elicited by high-frequency stimulations (HFS) consisting of three trains of 1 s at 100 Hz with 20 s intervals. Subsequent fEPSPs were recorded for more than 1 h post-HFS induction at the test pulse rate and intensity. Percent changes in the initial slope of the fEPSP before and after HFS were quantified.

### Hematoxylin and Eosin (HE) Staining

Morphological changes of cells in the hippocampal CA3-CA1 region were detected by HE staining. Briefly, the sections were deparaffinized in xylene for 15 min and rehydrated in descending grades of ethyl alcohol (100%, 95%, 85%, and 70%) and distilled water for 5 min each. Then, the sections were stained with 0.1% hematoxylin-eosin solution until the desired staining achieved, followed by being gradually dehydrated in 95% and 100% ethyl alcohol twice and 5 min each. After processing and dehydration, the slides were mounted with Eukitt Quick-hardening mounting medium and hippocampal neurons were observed under a light microscope (OLYMPUS 1X70-SIF2, Japan).

### Detection of Apoptosis by TUNEL

The TUNEL assay was performed to detect apoptosis. The paraffin-embedded hippocampus was sectioned at a thickness of 4 μm for TUNEL staining. After dewaxing, tissue sections were incubated with 3% hydrogen peroxide for 15 min and rinsed with PBS, then incubated with Protease K for 20 min and rinsed with PBS. After incubation with TUNEL assay mixture for 60 min and Peroxidase (POD) for 40 min, sections were treated with 3,3′-Diaminobenzidine (DAB), counterstained with Novocastra hematoxylin dye, dehydrated with alcohol, washed twice in xylene, and mounted. The positive cells detected by TUNEL were characterized as: cell nucleus aggregation, brown soma staining, chromatin condensation, and brownish yellow particles in the nucleus. The number of apoptotic cells restricted within the long-range of 1 mm in the middle of the hippocampal CA1 region was counted under the light microscope with 400× magnification. An apoptotic index (AI) was calculated as the number of TUNEL positive cells/total cells ×100.

### Western Blots

Western blotting assays were performed as described in a previous report (Cai et al., 2014). Hippocampal tissues were lysed using ice-cold radioimmunoprecipitation assay buffer (150 mM NaCl, 1% Triton X-100, 0.5% sodium deoxycholate, 1% SDS, 50 mM Tris–HCl, pH 8.0) supplemented with protease inhibitor mixture (Roche, Espoo, Finland). Equal amounts of protein (40 μg) were subjected to SDS-PAGE and blotted onto nitrocellulose filters. Blots were probed and recognized with the following primary and secondary antibodies: mouse anti-syntaxin (1:7,000), mouse anti-protein kinase C (PKC, 1:400), rabbit anti-protein kinase C-ζ (PKC-ζ, 1:500), rabbit anti-Gα(i); (1:1,000), rabbit anti-caspase-3 (1:1,000), mouse anti-β-tubulin (1:1,000), and HRP-labeled goat anti-rabbit or horse anti-mouse secondary antibody (1:1,000). Signals were detected by using enhanced chemiluminescence with ECL Western blotting detection reagent. The optical density (OD) of protein bands were analyzed using the BIO-RAD ChemiDoc™ Touch Imaging System (Bio-Rad, USA). The ODs for specific proteins were normalized to the corresponding β-tubulin levels, and these values were expressed as the fold relative to the control.

### Statistical Analyses

All data are presented as mean ± standard deviation (SD). The data were all normally distributed. Comparisons between the groups with different treatments were analyzed by analysis of variance (ANOVA), followed by the LSD or Dunnett’s T3 *post hoc* analysis. All statistical analyses were undertaken using the SPSS software (IBM Corp., Armonk, NY, USA). The significance level was set at 0.05 for all comparisons.

## Results

### CIH-Induced Cognitive Dysfunction Alleviated by A1R Signaling Activation but Exacerbated by Its Blockage

**Figure 1:**
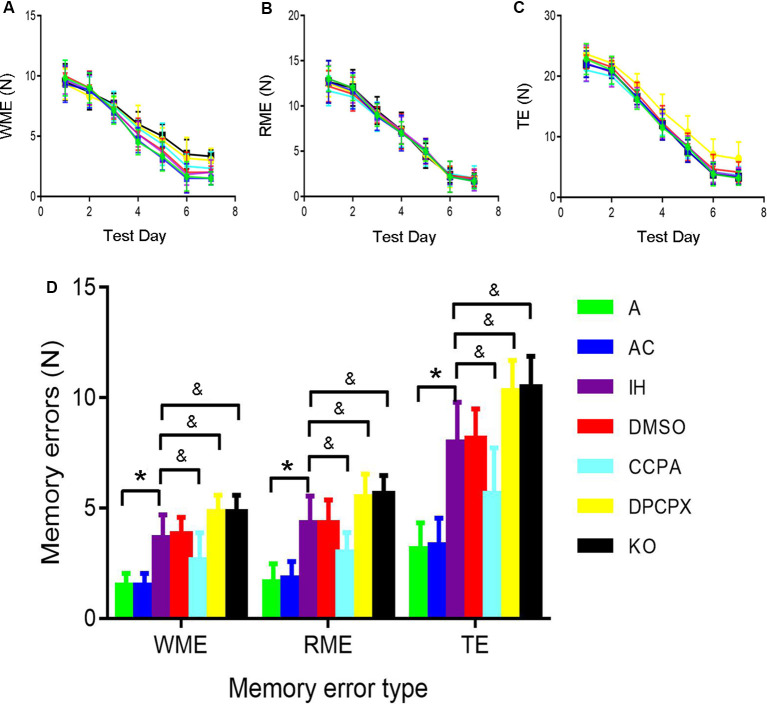
The 8-arm (4-arm baited) radial maze test in mice without or with treatments from air control groups (A and AC), IH exposed groups (IH and DMSO), CCPA-administrated group, DPCPX-administrated group, and *A1R* global knockout group (KO). ** (A–C)** The numbers of WME, RME, and TE on different training days, respectively. *N* = 6 mice per group. ** (D)** The numbers of memory errors of WME, RME, and TE in the testing session. *p ≤ 0.01 vs. A or AC; ^&^*p* ≤ 0.05 vs. IH or DMSO; *n* = 6 mice per group. Error bar: standard deviation. A, room air; AC, intermittent air; IH, intermittent hypoxia; DMSO, IH with DMSO treatment; CCPA, IH with 2-chloro-N(6)-cyclopentyl-adenosine treatment; DPCPX, IH with 8-cyclopentyl-1,3-dipropylxanthine treatment; A1R, adenosine A1 receptor; KO, IH with *A1R* knockout; WME, working memory errors; REM, reference memory errors; TE, total errors.

The 8-arm radial maze was used to measure spatial learning and memory capabilities. During the experiment, the mice did not appear to weight loss, exhibit any mental malaise, or die. We have made comparisons in the manuscript. Repeated-measures ANOVA showed that WME, RME, and TE in all groups significantly decreased throughout training sessions (Figures 1A–C). Furthermore, significant differences in the numbers of WME, RME and TE were present between groups at the same testing days after 6-day training (Figure 1D, RME: *F*_(6,35)_ = 17.675, *p* ≤ 0.01; WME: *F*_(6,35)_ = 17.368, *p* ≤ 0.01; TE: *F*_(6,35)_ = 24.596, *p* ≤ 0.01, *n* = 6 mice per group). A significant increase in RME, WME, and TE existed in the mice exposed to CIH for 4 weeks (RME: *p* ≤ 0.01 IH vs. A or AC group; WME: *p* ≤ 0.01 IH vs. A or AC group; TE: *p* ≤ 0.01 IH vs. A or AC group, *n* = 6 mice per group). However, the A1R agonist CCPA attenuated these CIH-induced cognitive deficits (RME: *p* ≤ 0.05 CCPA vs. IH or DMSO group; WME: *p* ≤ 0.05 CCPA vs. IH or DMSO group; TE: *p* ≤ 0.05 CCPA vs. IH or DMSO group, *n* = 6 mice per group), which were exacerbated by the A1R antagonist DPCPX or A1R gene deletion (RME: *p* ≤ 0.05 DPCPX or KO vs. IH or DMSO group; WME: *p* ≤ 0.05 DPCPX or KO vs. IH or DMSO group; TE: *p* ≤ 0.05 DPCPX or KO vs. IH or DMSO group, *n* = 6 mice per group). No statistical difference was detected between A and AC (RME: *p* = 0.763; WME: *p* = 1.000; TE: *p* = 0.849, *n* = 6 mice per group), IH and DMSO groups (RME: *p* = 1.000; WME: *p* = 0.730; TE: *p* = 0.849, *n* = 6 mice per group), or IH and KO groups (RME: *p* = 0.763; WME: *p* = 1.000; TE: *p* = 0.849, *n* = 6 mice per group).

### CIH-Induced Pathological Changes of Cellular Morphology in the Hippocampus

**Figure 2:**
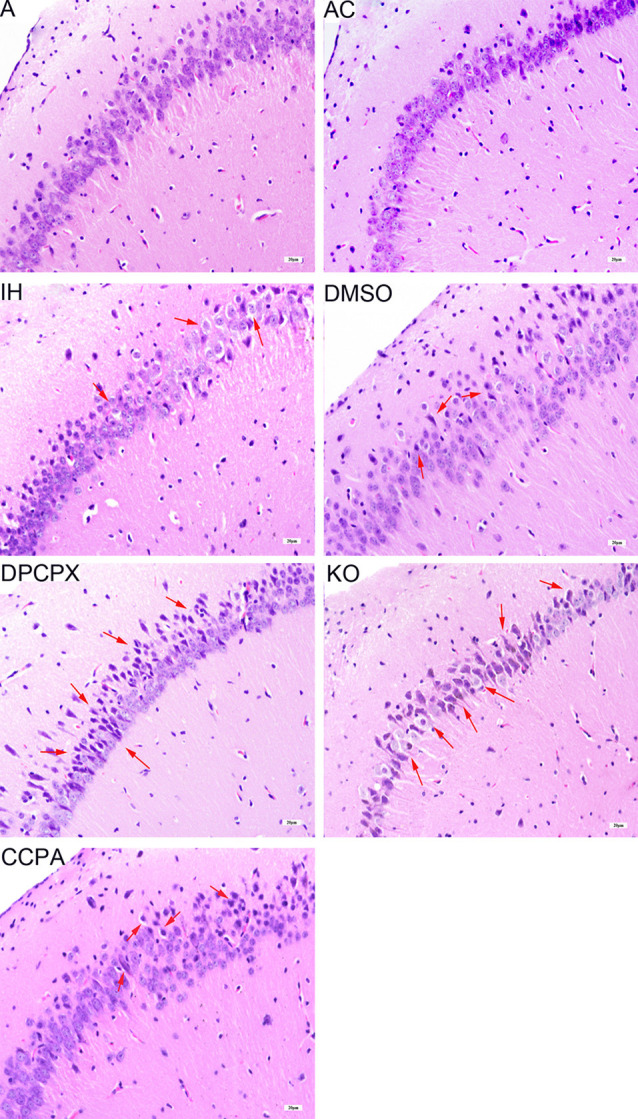
Neuronal morphology in the hippocampal CA3–CA1 region detected by hematoxylin and eosin staining. Arrows indicate swollen and heterologous neurons with clear nuclei. Bar: 20 μm. A, room air; AC, intermittent air; IH, intermittent hypoxia; DMSO, IH with DMSO treatment; CCPA, IH with 2-chloro-N (6)-cyclopentyl-adenosine treatment; DPCPX, IH with 8-cyclopentyl-1,3-dipropylxanthine treatment; KO, IH with *A1R* knockout.

To determine the pathological changes in the specific brain regions related to CHI-induced cognitive dysfunction, we focused on the hippocampal CA1 region using HE staining (Figure 2). The hippocampal neurons in the CA1 region were neatly arranged with normal size and shape in groups A and AC. The nuclear morphology was normal with a little heterochromatin and clear border of the nucleolus, mostly in the center of the cell body. In contrast, swollen and heterologous neurons with clear nuclei were visible in groups IH and DMSO (arrows) although the cellular size, shape, and structure were normal and intact. For the DPCPX group and KO group, heterologous and pyknotic neurons were distributed throughout the hippocampal CA1 region with blurred nuclei accompanied by anachronisms or even vacuolation. Compared with hippocampal neurons in the IH group, neurons in the CCPA group were neatly arranged while nuclear heterogeneity and cell swelling were reduced.

### CIH-Induced Apoptosis in Hippocampus Reduced by A1R Signaling Activation

**Figure 3:**
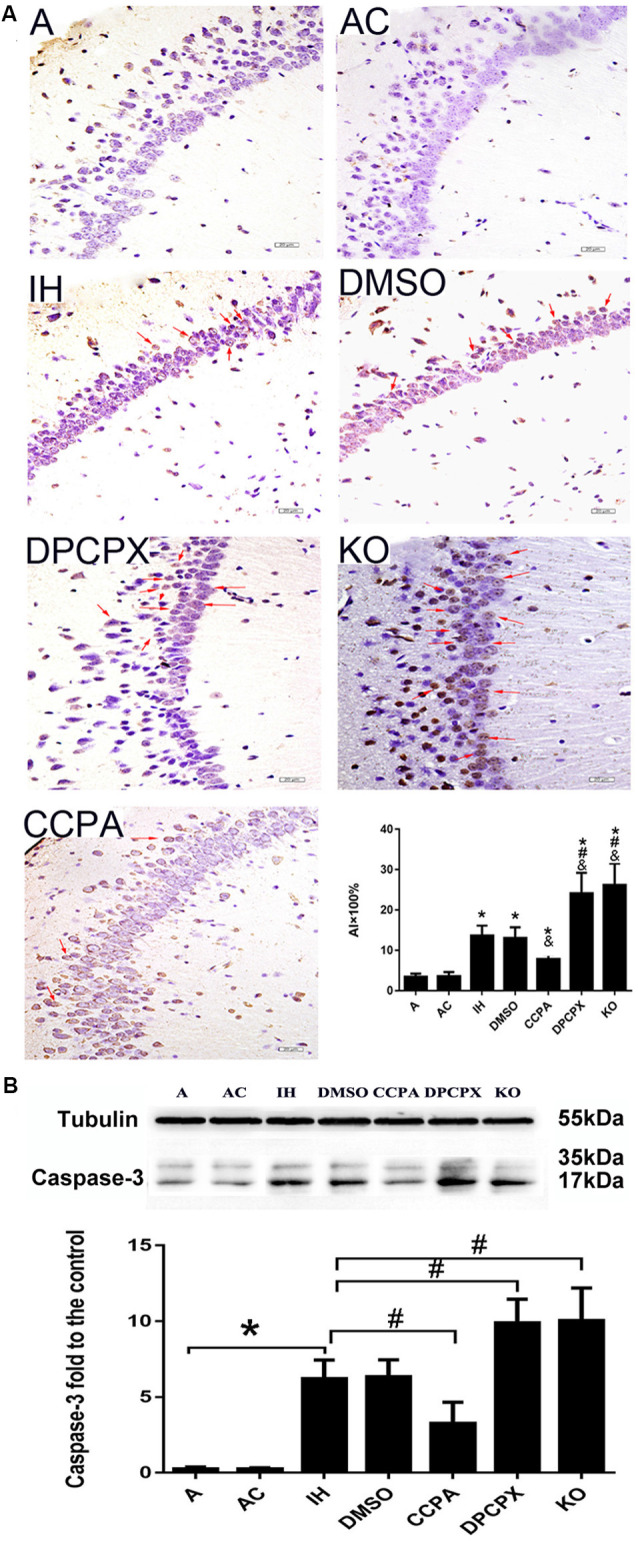
Neuronal apoptosis in the hippocampal CA3–CA1 region. ** (A)** Apoptotic neurons detected by TUNEL-DAB staining. Arrows indicate apoptotic neurons. *p ≤ 0.01 vs. A or AC; ^&^*p* ≤ 0.05 vs. IH or DMSO; ^#^*p* < 0.01 vs. CCPA; *n* = 6 mice per group. Bar: 20 μm. AI: apoptotic index. ** (B)** Relative levels ratio (vs. A) of cleaved caspase 3. *p ≤ 0.01 vs. A or AC; ^#^*p* ≤ 0.01 vs. IH or DMSO; *n* = 6 mice per group. A, room air; AC, intermittent air; IH, intermittent hypoxia; DMSO, IH with DMSO treatment; CCPA, IH with 2-chloro-N(6)-cyclopentyl-adenosine treatment; DPCPX, IH with 8-cyclopentyl-1,3-dipropylxanthine treatment; KO, IH with A1R knockout.

To further determine whether CIH results in cell apoptosis in the hippocampus, TUNEL-positive cells (arrows) were detected and the index of apoptosis was calculated by TUNEL staining (Figure 3A, *F*_(6,35)_ = 4.399, *p* ≤ 0.01, *n* = 6 mice per group). Compared to TUNEL-positive cells in mice of A or AC groups, more TUNEL-positive cells in the hippocampal CA3–CA1 region were observed in mice of all IH groups (red arrows). The apoptotic index (AI) was significantly different in all IH groups compared to that in A group or AC group (*p* ≤ 0.01 vs. A or AC). Compared with the AI in IH or DMSO group, AI was significantly increased in both the DPCPX group and the KO group (*p* ≤ 0.05 vs. IH or DMSO), whereas AI in the CCPA group was much lower than that in the IH or DMSO groups (*p* ≤ 0.05 vs. IH or DMSO). There was no statistically significant difference between A and AC groups (*p* = 0.940) or between IH and DMSO groups (*p* = 0.753). These results were corroborated by increased levels of cleaved caspase 3 (Figure 3B).

### Activation of A1R Signaling Prevents CIH-Induced Decline of Hippocampal LTP

**Figure 4:**
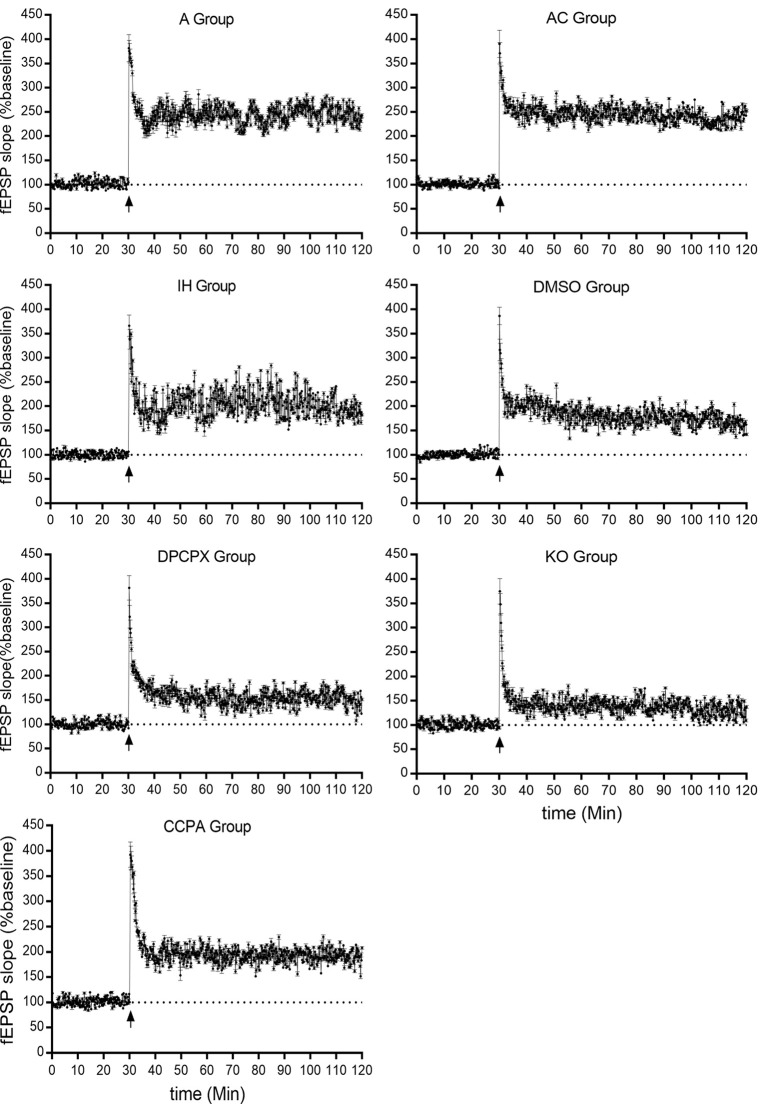
Hippocampal LTPs in mice without or with treatments from air control groups (A and AC), IH exposed groups (IH and DMSO), CCPA-administrated group, DPCPX-administrated group, and *A1R* global knockout group (KO). LTP, long term potential; fEPSP, field excitatory postsynaptic potential; A, room air; AC, intermittent air; IH, intermittent hypoxia; DMSO, IH with DMSO treatment; CCPA, IH with 2-chloro-N (6)-cyclopentyl-adenosine treatment; DPCPX, IH with 8-cyclopentyl-1,3-dipropylxanthine treatment; KO, IH with *A1R* knockout.

LTP represents a long-lasting increase in synaptic strength, which is considered one of the major cellular mechanisms that underlie learning and memory. To determine whether CIH affects synaptic plasticity, LTP was measured in the CA3–CA1 region of the hippocampus. Significant differences were observed in hippocampal LTP among the experimental groups (Figure 4 and Table 1, *F*_(6,35)_ = 70.61, *p* < 0.01, *n* = 6 mice per group). The magnitude of LTP in IH (increase by 68.45 ± 15.36% compared to baseline) and DMSO (increase by 62.92 ± 11.07% compared to baseline) groups were much lower than those in A (increase by 142.58 ± 2.91% compared to baseline) and AC (increase by 139.14 ± 10.99% compared to baseline) groups (*p* < 0.01 vs. A or AC) which were dramatically exacerbated by the A1R antagonist DPCPX (increase by 39.88 ± 15.38% compared to baseline) or A1R gene disruption (increase by 37.12 ± 6.20% compared to baseline; *p* < 0.01 vs. IH or DMSO). However, A1R agonist CCPA (increase by 95.44 ± 11.69% compared to baseline) prevented the decline of hippocampal LTP from CIH exposures (*p* < 0.01 vs. IH or DMSO). Differences between A and AC groups (*p* = 0.643) or between IH and DMSO groups (*p* = 0.453) were not significant.

**Table 1:** The long-term potentials in hippocampal CA3–CA1 region.

Groups	Number of independent experiments	LTP
A	6	142.58 ± 2.91
AC	6	139.14 ± 10.99
IH	6	68.45 ± 15.36*
DMSO	6	62.92 ± 11.07
CCPA	6	95.44 ± 11.69^&^
DPCPX	6	39.88 ± 15.38^&^
KO	6	37.12 ± 6.20^&^
F		70.61
*P*		<0.01

Data were presented as mean ± SD. *p < 0.01 vs. A or AC; and ^&^*p* < 0.01 vs. IH or DMSO; *n* = 6 slices of three mice per group. LTP, long-term potential; A, room air; AC, intermittent air; IH, intermittent hypoxia; DMSO, IH with DMSO treatment; CCPA, IH with 2-chloro-N (6)-cyclopentyl-adenosine treatment; DPCPX, IH with 8-cyclopentyl-1,3-dipropylxanthine treatment; KO, IH with *A1R* knockout.

### Downstream Molecules PKC, PKC-ζ, Gα(i), and Syntaxin Responding to Modulation of A1R Signaling in the Hippocampus After CIH Exposures

**Figure 5:**
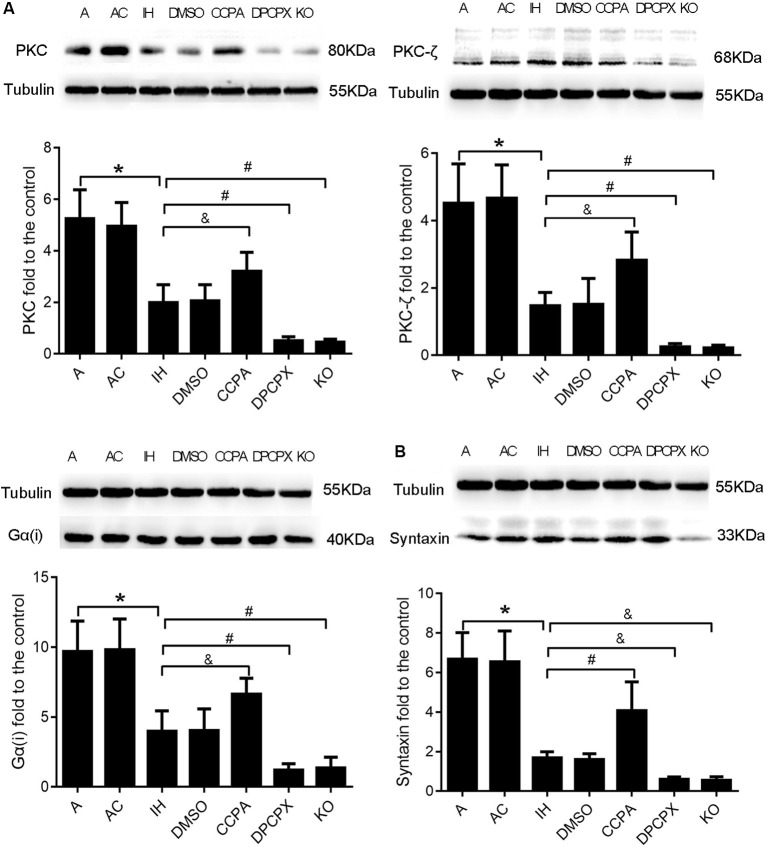
Levels of A1R downstream molecules PKC, PKC-ζ, Gα(i), and syntaxin in hippocampi dissected from mice without or with treatments from air control groups (A and AC), IH exposed groups (IH and DMSO), CCPA-administrated group, DPCPX-administrated group, and A1R global knockout group (KO). ** (A)** Relative Levels ratios (vs. A) of A1R downstream signaling PKC, PKC-ζ, and Gα(i). ** (B)** Relative Levels ratio (vs. A) of synaptic transmission-regulatory membrane protein—syntaxin. *p ≤ 0.01 vs. A or AC; ^#^*p* ≤ 0.01 vs. IH or DMSO; ^&^*p* ≤ 0.05 vs. IH or DMSO; *n* = 6 mice per group. A, room air; AC, intermittent air; IH, intermittent hypoxia; DMSO, IH with DMSO treatment; CCPA, IH with 2-chloro-N (6)-cyclopentyl-adenosine treatment; DPCPX, IH with 8-cyclopentyl-1,3-dipropylxanthine treatment; A1R, adenosine A1 receptor; KO, IH with *A1R* knockout.

To further confirm the neuroprotective effects of A1R signaling on cognitive impairment induced by CIH, we examined the levels of four downstream proteins—PKC, PKC-ζ, Gα(i), and syntaxin in the hippocampus after CIH (Okada et al., 2001; Di-Capua et al., 2003; Crawford et al., 2011; Rombo et al., 2016). Levels of PKC, PKC-ζ, Gα(i), and syntaxin were significantly different in all experimental groups (Figure 5, PKC: *F*_(6, 63)_ = 4.320, *p* ≤ 0.01; PKC-ζ: *F*_(6,63)_ = 4.947, *p* ≤ 0.01; Gα(i): *F*_ (6,63)_ = 4.725, *p* ≤ 0.01; syntaxin: *F*_(6,63)_ = 14.187, *p* ≤ 0.01; *n* = 6 mice per group). Consistent with changes of cognition, apoptosis, and LTP in hippocampal regions, levels of PKC, PKC-ζ, and Gα(i) were significantly declined while levels of syntaxin were dramatically up-regulated in IH and DMSO groups (PKC: *p* ≤ 0.01; PKC-ζ: *p* ≤ 0.01; Gα(i): *p* ≤ 0.01; syntaxin: *p* ≤ 0.01; IH or DMSO vs. A or AC), and more severe change was observed in DPCPX and KO groups (PKC: *p* ≤ 0.01, PKC-ζ: *p* ≤ 0.01, Gα(i): *p* ≤ 0.01 vs. IH or DMSO; syntaxin: *p* ≤ 0.05 vs. IH or DMSO). The altered levels were partially rectified when the A1R agonist CCPA was administrated (PKC: *p* ≤ 0.05, PKC-ζ: *p* ≤ 0.05, Gα(i): *p* ≤ 0.05 vs. IH or DMSO; syntaxin: *p* ≤ 0.01 vs. IH or DMSO).

## Discussion

### OSAHS-Featured Chronic Intermittent Hypoxia-Induced Pathological and Functional Changes in the Hippocampus

Learning and memory are fundamental brain functions and different brain areas play different roles in this process (Benfenati, 2007). Extensive studies have shown that intermittent hypoxia induces damage in the frontal cortex and hippocampal CA1 area (Goldbart et al., 2003; Row et al., 2003). Our study used an eight-arm maze and a modified computer-controlled CIH model to confirm the adverse effect of CIH on learning and memory function in mice. The results showed that the reference RME, WME, and TE were gradually reduced with training. Following the learning and memory processes, these data quantify the formation of spatial memory throughout training (Figures 1A–C). CIH exposures significantly increased RME, WME, and TE consistent with learning and memory impairment in the mice (Figure 1D).

Working memory is a form of short-term memory. After a certain period of training, spatial reference memory has changed from short-term memory into long-term memory. The hippocampus mediates this process. Hippocampus and prefrontal cortex are the main anatomical locations of learning and memory (Zheng and Zhang, 2015), and the hippocampal CAl region is associated with spatial memory (Mehta, 2015). We found CIH caused neuronal damage in the hippocampus, apparent in degenerative changes in morphology and increased apoptosis (Figures 2, 3).

In the nervous system, neurons contact each other by synapses to form neuronal networks (Flores et al., 2016). These networks undergo a constant transformation in response to the activity *via* mechanisms of synaptic plasticity (Amtul, 2015). One of the most extensively studied forms of synaptic plasticity is LTP and long-term depression (LTD) in the hippocampus. They can modify and regulate the synapses, which are believed to be essential processes for learning and memory. LTP also enhances synaptogenesis in the developing hippocampus and enhances structural synaptic plasticity (Hohoff et al., 2014). Previous studies demonstrated that CIH during the light phase impairs LTP in the hippocampal CA1 but not in the dentate gyrus of rat (Wang et al., 2001; Payne et al., 2004; Wall et al., 2014). Payne et al. (2004) found that CIH can disturb the excitability of hippocampal neurons and inhibit the levels of BDNF, leading to a loss of the magnitude of LTP and memory deficits. PS-LTP induction and sustenance *in vitro* can be used as a quantitative measure of cognitive damage (Payne et al., 2004). It was also reported that CIH does not affect pair-pulse facilitation but suppress LTP which can be reversed with BDNF treatment, suggesting a postsynaptic mechanism (Xie et al., 2010). Most recently, Khuu et al. (2019) found that CIH induces both ROS-dependent and ROS-independent effect on adult neurogenesis and synaptic plasticity in the dentate gyrus, which can be mitigated by antioxidant treatment, indicating that oxidative signaling caused by CIH is a significant factor that impairs synaptic plasticity in the hippocampus (Khuu et al., 2019). Consistently, in the present study, 4-week CIH exposures blunted the LTP at CA3-CA1 hippocampal synapses (Figure 4 and Table 1) which likely underlies the cognitive impairment (Figure 1). CIH has previously been shown to impair synaptic plasticity, specifically LTP. However, the underlying mechanism of IH on synaptic plasticity is rarely known.

### Neuroprotection *via* A1Rs Signaling on CIH-Induced Cognitive Dysfunction and Pathological Changes in the Hippocampus

Previous studies showed that adenosine accumulates under hypoxia (Rubio et al., 1975; Winn et al., 1981) and hypoxia-induced synaptic depression is mediated by the presynaptic neuromodulator adenosine (Lucchi et al., 1996; Dunwiddie and Masino, 2001; Gervitz et al., 2001; Brust et al., 2006; Mukandala et al., 2016). Activation of A1Rs demonstrated neuronal protection in the rodent model of acute hypoxia or ischemia-reperfusion (Zamani et al., 2013; Tregub et al., 2014). The detrimental effects of A1R activation on neuronal cells, cognition, and synaptic plasticity were also found in cultured PC12 cells and other neurological diseases such as Alzheimer’s disease and Parkinson’s disease (Chen, 2014; Giust et al., 2014; Mei et al., 2018). The Possible reason is long-time exposures under hypoxia cause a desensitive adenosine response. However,* in vivo* functional response of A1Rs signaling to the OSAHS-featured CIH has not yet investigated. In this study, A1R agonist- or antagonist-administrated mice and conventional A1R gene knockout mice were exposed to CIH to determine the precise function of A1R signaling. We found that CIH-induced morphological damage and apoptosis in hippocampal neurons were aggravated by A1R antagonist or A1R gene deletion but partially rescued when A1R signaling was activated by the agonist (Figures 2, 3), correlating to the functional change of learning and memory (Figure 1). Taken together, these results provided strong evidence that activation of A1R signaling protects hippocampal neurons and cognitive function against CIH insult.

### Potential Mechanism (s) Underlying the Neuroprotection of A1Rs Signaling in CIH-Induced Cognitive Impairments

Adenosine, as an important central modulator, is widely distributed in the tissues and organs of the human body. It modulates synaptic plasticity by binding to the inhibitory A1 and facilitatory A2A receptors. Adenosine pronouncedly modulates hippocampal LTP *via* activation of A2AR rather in aged than young animals. However, the selective A1R antagonist DPCPX increased LTP and that effect tends to be more pronounced in young than in aged rats. Suggesting that adenosine only regulates hippocampal LTP through A1R activation in young animals (Costenla et al., 2011). In adult animals, A2AR mediates the effects of adenosine on memory dysfunction (Li P. et al., 2015; Laurent et al., 2016; Silva et al., 2016) which is sufficient to cause a deficit of reference memory (Li P. et al., 2015). Thus, A1R and A2AR may play diverse roles in OSAHS-induced cognitive impairments, which could explain why caffeine (antagonist on both A1R and A2AR) consumption does not change OSA-induced cognitive dysfunction (Costenla et al., 2011; Pinheiro et al., 2014). In our study, we established a model of OSAHS in young mice and demonstrated that A1R activation controls synaptic plasticity and memory performance.

Adenosine A1Rs are widely distributed in the brain and mediate information transmission through the downstream Gα(i)-cAMP-PKC pathway. Many studies have shown that PKC and its subtypes play an important role in maintaining LTP and synaptic plasticity (Besalduch et al., 2013; Jalil et al., 2015; Li C. et al., 2015). It was also reported that PKC is upregulated after chemical LTP induction (Palida et al., 2015). Here we demonstrated that the levels of Gα(i), PKC, and its subtype PKC-ζ were significantly suppressed by CIH. The suppression was exacerbated *via* partial or complete blockade of A1R signaling but relieved *via* A1R activation (Figure 5A). These data suggest that A1R signaling modulation is mediated by Gα(i)-cAMP-PKC pathway.

Previous studies have shown that neuronal activity in the hippocampus is regulated by the major pathway of N-type voltage-sensitive Ca^2+^ channels/calcium-phospholipid-dependent PKC/syntaxin and the minor pathway of P-type voltage-sensitive Ca^2+^ channels/cyclic AMP-dependent protein kinase (PKA)/synaptobrevin (Okada et al., 2001). Syntaxins are a family of membrane-integrated proteins at presynaptic active zones that may mediate Ca^2+^-triggered synaptic vesicle exocytosis. Loss of syntaxins results in the impaired neurotransmitter release and affects synaptic transmission as well as neural function (Quick, 2006; Shin, 2014). In our study, the levels of hippocampal syntaxin protein in different experimental groups supported this observation (Figure 5B)—the less levels of syntaxin in the hippocampus, the worse cognitive performance. Syntaxin-1 is not only highly expressed at the presynaptic active zone but also colocalized postsynaptically within the nanometer range with NMDA receptor subunit NR2B, suggesting that syntaxin-1 may be involved in NR2B vesicular trafficking and enhancing learning and memory *via* facilitating synaptic plasticity (Hussain et al., 2016). Thus, further analysis is imperative to assess the role of A1Rs in both pre- and post-synaptic function underlying the CIH-induced cognitive deficits.

OSAHS is a breathing disorder that is associated with cognitive impairment. While the profile of cognitive impairment in OSAHS is becoming clearer, the mechanisms of OSAHS-induced cognitive deficits remain unknown. The predominant theoretical framework of cognitive impairment focuses on hypoxia, hypercarbia, and sleep disruption (Beebe and Gozal, 2002; Beebe, 2005). The present study demonstrated that CIH induces morphological changes and apoptosis in hippocampal neurons, leading to cognitive dysfunction. Activation of adenosine A1R signaling plays a neuroprotective role and modulates the downstream Gα(i)-cAMP-PKC pathway, thus promoting the formation of LTP and enhancing synaptic plasticity as well as learning and memory.

One limitation of the present study is that we did not control the parameters of the peripheral system. Whereas A1R is well established to modify several peripheral systems, namely body temperature, cardiovascular parameters, respiratory parameters, and consequently biochemical parameters (pH, pCO_2_, pO_2_), all of which can impact indirectly on brain function. Our future studies will explore if the presently reported impact of A1R on cognitive impairment induced by CIH might indirectly result from an A1R-mediated control of any of these parameters.

Intense efforts by many pharmaceutical companies and academicians in the A1R agonist field have entered clinical trial stages. However, the use of A1R agonists has been met by limited success due to the cardiovascular side effects and well-defined desensitization in both animal models and human trials. Nonetheless, the reduction in CIH-induced cognitive impairment *via* A1R agonism may have long-awaited clinical success soon.

## Data Availability Statement

All datasets included in this study are available upon request from the corresponding author.

## Ethics Statement

The animal study was reviewed and approved by Wenzhou Medical University.

## Authors Contributions

XC, JC, YZ, and HC conceived and designed the experiments. YZ, HC, XC, DX, and XQ performed the experiments. YZ, HC, DX, and XQ conducted the animal study. YC, GB, YT, ATG, and AHG analyzed the data. XC, JC, YZ, and HC supervised the experiments. YZ and HC drafted the manuscript. XC, JC, CP, and GB revised the manuscript. All authors approved the final version of the manuscript.

## Conflict of Interest

The authors declare that the research was conducted in the absence of any commercial or financial relationships that could be construed as a potential conflict of interest.
